# The Gender Award Gap in German medical societies 2000–2023: the Fritz-Külz-Award as an example

**DOI:** 10.1007/s00210-025-03892-8

**Published:** 2025-03-06

**Authors:** Thorsten Halling, Viola Mambrey, Jessica Marie Steinert, Roland Seifert, Annegret Dreher, Chantal Marazia, Adrian Loerbroks, Nils Hansson

**Affiliations:** 1 Department for the History, Philosophy and Ethics of Medicine, Center for Health and Society, Medical Faculty, Heinrich Heine University Düsseldorf, Düsseldorf, Germany; 2 Institute of Occupational, Social, and Environmental Medicine, Center for Health and Society, Medical Faculty, Heinrich Heine University Düsseldorf, Düsseldorf, Germany; 3 Department of Pharmacology, Hannover Medical School, Hannover, Germany; 4 Department of Clinical Sciences and Community Health, University of Milan, Milan, Italy

**Keywords:** Prizes, Gender equity, Pharmacology, Recognition, Prestige, Credit

## Abstract

Science prizes contribute to the visibility of researchers within and outside the medical community. Our article contains a descriptive analysis of the prize development in German medical societies since the turn of the millennium, focussing on the development of gender differences and discussing the findings with regard to necessary structural changes and the general significance of prizes in medicine. The study is based on data from all documented prizes and honours awarded by the 183 German medical societies currently organised in the Association of the Scientific Medical Societies in Germany (AWMF, Arbeitsgemeinschaft der Wissenschaftlichen Medizinischen Fachgesellschaften) in the period 2000–2023, including the Fritz-Külz-Award in pharmacology. For the first time, our study enables a differentiated overall view of the diverse prize culture in German medical societies, with 1213 awards (including 201 scholarships). The results show that the gender award gap found in international studies, particularly for prestigious awards, has continuously narrowed in the awarding practice of German medical societies since 2000. However, a gender-specific imbalance is still recognisable, particularly in the case of prestigious honorary prizes and more highly endowed research prizes. Differences between the specialist societies, depending on the speciality, the respective proportion of female specialists and the proportion of female scientists among the members must be investigated in further detailed studies. The specialist societies are therefore called upon to make their nomination and application practices even more transparent in order to better recognise potential disadvantages.

## Introduction

Recognition in the sciences is primarily expressed in the form of project funding, citations, academic positions, memberships and functions in scientific committees and prizes. These elements unfold their value for researchers in particular in their interplay. However, unlike citations and funding, which can be quantified precisely, prizes have so far eluded clear categorisation in scientific evaluation systems (Hansson and Angetter-Pfeiffer 2021, Hansson and Schlich 2024).

Nevertheless, science prizes make a decisive contribution to the visibility of researchers and for a long time also to the invisibility of female researchers within and outside the scientific community. To date, less than 7% of science Nobel Prizes have gone to women (Widmalm 2018). In more recent international studies, a cross-national and cross-disciplinary selection of prestigious awards was used to emphasise the discrimination of female scientists in the awarding of prizes—even beyond the Nobel Prize (Malik et al. 2024, Lagisz et al. 2023, Pohar and Hansson 2020). In an analysis covering the period 1968–2017 (*N* = 628 prizes), 11.3% of laureates were female researchers (Ma et al 2019). On average, women receive fewer research prizes and honorary prizes for their life time achievements (Martin et al. 2020; Fang et al. 2021; Atkinson et al 2019) and are more likely to be honoured for teaching activities and involvement in professional societies (Lincoln et al 2012). This also means that female researchers are less likely to receive highly endowed prizes. The slow progress in overcoming this disadvantage is documented by the analysis of 141 prestigious science prizes—also across countries and disciplines—in the years 2016–2020. 19% of these prizes went to women and this proportion is lower than the proportion of female professors in the disciplines analysed (Meho 2021). These observations were also made by Anglo-American studies in various medical disciplines, e.g., in anaesthesiology (Ellinas et al 2019), dermatology (Shukla et al 2019), neurology (Martindale et al. 2004), emergency medicine (Fang et al 2021), orthopaedics (Gerull et al. 2021), rehabilitation medicine (Silver et al. 2018) and rheumatology (Halling et al 2022).

Even though university medicine is becoming increasingly female, the term ‘leaky pipeline’, which describes the decline in the proportion of women with each additional qualification level, is still justified today (Ferry 2020). In the search for the causes, two arguments were discussed in prize research, which are also cited for other phenomena of discrimination against women in the sciences (e.g. gender citation gap, gender career gap, and gender pay gap): Homophily and the so-called ‘Matilda effect’. In relation to prices, homophily refers to the tendency of individuals to favour people similar to themselves in certain situations, e.g. that men tend to choose men and women tend to choose women (Gallotti and Domenico 2019). An earlier study has shown that the proportion of women, and in particular a female chairperson of a prize committee, increases the probability that a female scientist will be honoured (Lincoln et al 2012). The authors supplement this finding for their data on prize awards in the 1990s and 2000s in the USA with the so-called Matilda effect, the suppression of contributions by female scientists whose research achievements are attributed to their male colleagues (Rossiter 1993).

As part of our three-year project ‘Gender Award Gap—(In)Visibility of Women in the Recognition Cultures of Medicine’ from 2021 to 2024, a systematic analysis of these issues was carried out for German medical societies.

For the first time, our study enables a differentiated overall view of a diverse award culture in medicine, with more than a thousand awards from over 180 specialist societies. The term ‘prize culture’ describes the formulated objectives in the context of the general tasks of the professional societies and the verifiable practice with regard to the awards presented (including prize categories, application requirements, selection process, endowment and funding, prize winners). For example, festive award ceremonies are an integral part of the annual congresses of the professional societies. They recognise individuals who have rendered outstanding services to the development of the respective specialist field and scientific work that is defined as outstanding. This article focuses on the quantitative development of prizes and honours in the period 2000-2023 and the question of the extent to which the ‘gender award gap’ that has been demonstrated for international specialist societies can also be observed in Germany. As an example, we analysed the Fritz-Külz-Award of the German Society for Experimental and Clinical Pharmacology and Toxicology (Deutsche Gesellschaft für Experimentelle und Klinische Pharmakologie und Toxikologie, DGPT) in more detail.

## Materials and methods

The data basis of the study is formed by all documented prizes and honours awarded by the 183 German medical societies currently organised in the Association of the Scientific Medical Societies in Germany (AWMF). This also includes scholarships, some of which also operate as prizes and are only recognisable as scholarships on the basis of the respective statutes. Information on the specialist societies, the prizes and award procedures as well as the prizewinners was systematically recorded for the period 2000–2023. The sources used were the corresponding call texts and lists on the websites of the scientific societies and additionally in their press releases and subject-specific as well as general publications. Comprehensive data was available for 178 out of 183 scientific societies. Missing information was requested by e-mail from the respective offices, with a reminder if necessary. Complete or partial information was provided by 98 of the 172 specialist societies contacted. As not all data was available from the specialist societies themselves, particularly for the first decade of the study period, the individual analyses each include different subsets of the above-mentioned population. The following variables were analysed descriptively: award period, award category, number and type of award winners (researcher/researcher/team) per year, and endowment. The data could not be correlated with the total number of female medical specialists in the speciality or with the proportion of female researchers among members of the respective specialist society, firstly because many prizes are awarded across disciplines (i.e. also for basic scientists) and secondly because gender-specific membership statistics are only available selectively or cannot be accessed for data protection reasons.

## Results and discussion

### Prize cultures

**Fig. 1:**
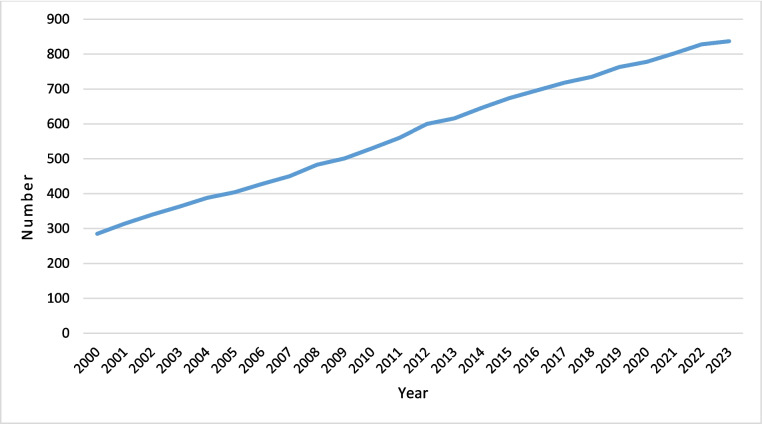
Number of prizes and honours awarded by medical societies in Germany 2000–2023

During the period under review, the German medical societies organised in the Association of the Scientific Medical Societies in Germany (AWMF) awarded at least 1213 prizes and honours (including 201 scholarships). The award period is documented for 69% of the prizes and honours (*N* = 873). In this sub-corpus, the number of prizes has almost tripled (see Fig. 1). A continuous, almost linear development is recognisable. Every year, 20 to 30 more prizes are awarded in absolute terms.

The prize cultures of the specialist societies differ considerably in terms of the number of awards presented, the awarding rhythm, prize categories, application modalities, sponsors and tradition.

#### Number of awards and awarding rhythm

Fifty-three percent of the specialist societies awarded up to five, 39.5% up to 20 and 4.9% more than 20 prizes and honours in the period under review, with only 61.6% of the awards being presented annually. Only five specialist societies (2.7%) did not award any prizes. The German Society for Cardiology, Cardiovascular Research stands out with a total of 65 awards.

#### Award categories

From the spectrum of honoured achievements and specific forms of presentation of scientific research in medicine (including lecture, poster or film awards), which has become increasingly differentiated in recent years, six main categories were formed for the present evaluation and their shares calculated (*N* = 1213): Research prizes (62.9%), scholarships/research funding (16.6%), honour prizes (13.3%), patient care (3%), teaching (1.7%) and media prizes/science communication (1.6%). Twelve prizes (1%) that are no longer awarded could not be allocated due to a lack of information.

#### Application modalities

An active application is required for most awards, especially for research prizes (58%). In contrast, the honorary prizes in particular are generally only awarded on nomination and are usually associated with a representative medal (without prize money). In the case of abstract, lecture, poster and ‘best paper’ prizes, the respective submission included participation in the selection process (15.5%). No information was available for 10.4% of the prizes. In addition to professional qualifications, the group of applicants was and is formally limited by various forms of age restrictions for 514 of the 1213 prizes (42.4%). In addition to criteria that are not clearly defined (e.g. ‘early career researcher’ or ‘early career award’) (16.4%), these include biological age (15.5%) and the maximum distance between the completion of academic qualifications (e.g. dissertation or habilitation) (8.2%) and academic qualifications (e.g. dissertation or habilitation) (8.2%). Only in the admission regulations of 26 awards (2.1%) is explicit reference made to the possibility of having periods of parental leave or other care periods recognised.

#### Endowment and sponsors

**Fig. 2:**
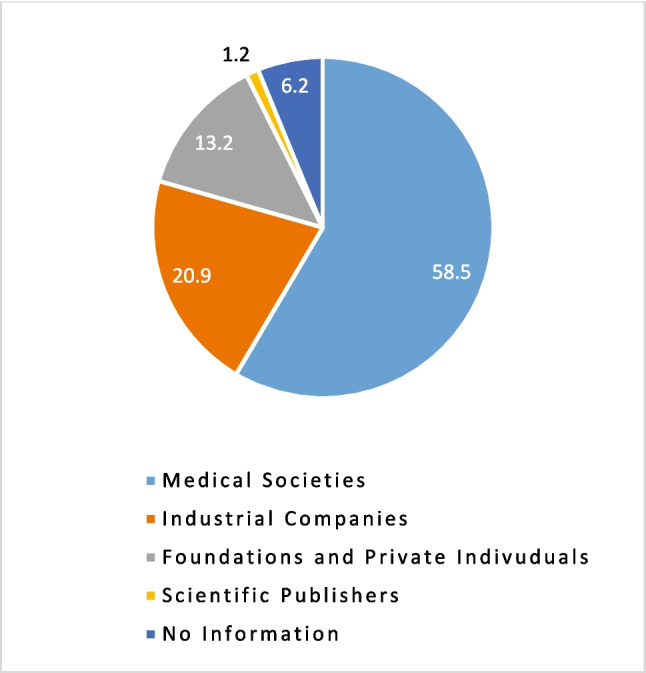
Funding and sponsoring of Scientific Awards. Data are shown as percentages in a pie chart

Important indicators for the significance of individual prizes in the specialist societies are the endowments associated with them, but also their tradition. The vast majority of the endowed prizes and scholarships (*N* = 700) include prize money of 1000–4999 euros (28.9%). However, many of the endowed prizes were not or not exclusively funded by the scientific societies themselves, but also by industrial companies, foundations and private individuals and scientific publishers (see Fig. 2).

In 2023, the total amount of prizes endowed and actually awarded this year (*N* = 463) totalled 2,988,300 euros, including scholarships (*N* = 513) 4,144,300 euros.

#### Tradition

In line with the large increase in prizes over the last two decades (see Fig. 1), only 350 (28.9%) of the prizes—for which the year of the first award could be determined (*N* = 946)—have been awarded for more than 20 years. Sixty-three prizes (5.9%) can point to a tradition of more than 50 years. Some are even considerably older than the Nobel Prize (since 1901): The ‘von Graefe Prize’ of the German Ophthalmological Society was awarded for the first time as early as in 1876.

### Award winners

The following results relate to a subset of awards for which complete data on the respective award winners and research teams could be determined for the entire period under review. A possible gender award gap was investigated with the help of descriptive analyses. To this end, the proportion of female award winners was compared to the proportion of male award winners and teams with regard to the distribution in the award categories and the prize money.

**Fig. 3:**
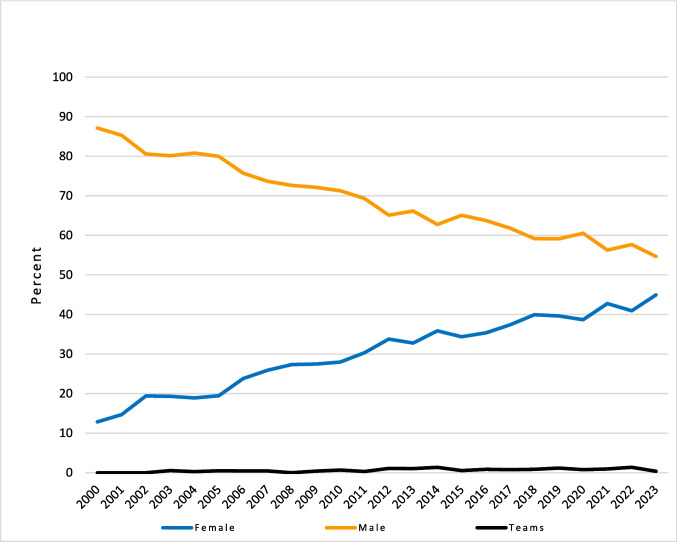
Prizes and honours awarded by medical societies in Germany 2000–2023 by year and award winner (in percent)

A continuous equalisation process can be observed during the period under review, so that the gender award gap has been reduced to a few percentage points in relation to the awarding of prizes and honours by medical societies in Germany (Fig. 3, Table 1).

**Table 1:** Prizes and honours awarded by medical societies in Germany 2000–2023 by year and award winner

Year	Awards	Awardees (total)	Female	%	Male	%	Teams	%
2000	193	280	36	12.86	244	87.14	0	0.00
2001	208	306	45	14.71	261	85.29	0	0.00
2002	231	340	66	19.41	274	80.59	0	0.00
2003	251	352	68	19.32	282	80.11	2	0.57
2004	266	365	69	18.90	295	80.82	1	0.27
2005	276	385	75	19.48	308	80.00	2	0.52
2006	300	437	104	23.80	331	75.74	2	0.46
2007	312	456	118	25.88	336	73.68	2	0.44
2008	337	483	132	27.33	351	72.67	0	0.00
2009	340	506	139	27.47	365	72.13	2	0.40
2010	375	568	159	27.99	405	71.30	4	0.70
2011	400	600	182	30.33	416	69.33	2	0.33
2012	410	648	219	33.80	422	65.12	7	1.08
2013	433	662	217	32.78	438	66.16	7	1.06
2014	439	666	239	35.89	418	62.76	9	1.35
2015	482	727	250	34.39	473	65.06	4	0.55
2016	491	806	285	35.36	514	63.77	7	0.87
2017	498	796	298	37.44	492	61.81	6	0.75
2018	527	818	327	39.98	484	59.17	7	0.86
2019	562	913	362	39.65	540	59.15	11	1.20
2020	394	636	246	38.68	385	60.53	5	0.79
2021	504	837	358	42.77	471	56.27	8	0.96
2022	517	867	355	40.95	500	57.67	12	1.38
2023	470	823	370	44.96	450	54.68	3	0.36

**Fig. 4:**
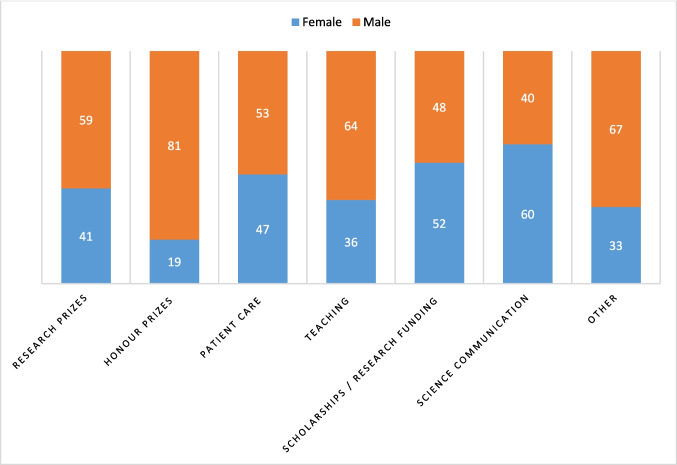
Prizes and honours awarded by medical societies in Germany 2000–2023 by prize category and award winner. Data are shown as percentages

Despite this levelling, there are also clear differences in the gender distribution (i.e. proportion of female award winners vs. proportion of award winners/teams for the years 2019–2023) for the last five years covered (2019–2023), differentiated according to the main categories of the award types (Fig. 4). The slightly above-average proportion of female award winners in the patient care category and the significantly above-average proportion of female award winners in the scholarships/research funding and media awards/science communication categories contrasts with the significantly below-average proportion of female scientists in the case of honorary prizes, which are awarded for a lifetime achievement.

**Fig. 5:**
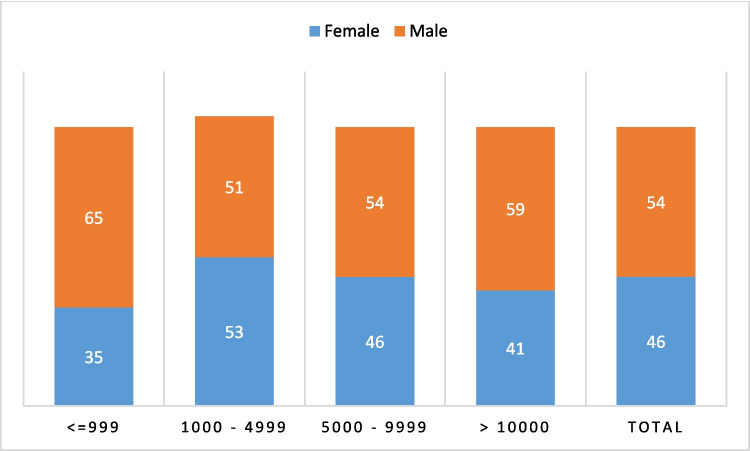
Awards by endowment and gender (2023). Prize amounts are given in €

In contrast to the prize categories, the differences between the awards in terms of endowment and gender (for 2023) are significantly smaller (Fig. 5).

## Prizes in pharmacology: gender distribution of the recipients of the Fritz-Külz-Award

We analysed the gender distribution of the winners of the Fritz-Külz-Award of the German Society for Experimental and Clinical Pharmacology and Toxicology (DGPT). We identified 45 prize winners from 1969 to 2023 (the Fritz-Külz-Award was not awarded from 2014 until 2018); 28 were male (62.22%) and 17 female (37.78%) (Table 2).

**Table 2:** List of prize-winners of the Fritz-Külz-Award

Year of award	Recipient
1969	Hans Winkler
1972	Ulrich Abshagen
1974	Volker Höllt
1974	Klaus Turnheim
1976	Walter E. Müller
1976	Thomas Schwartzkopff
1978	Michael Wüster
1980	Wolfgang Legrum
1980	Ursula Havemann
1982	Dietmar Trenk
1982	Thomas Simmet
1984	Ulrich Beuers
1984	Bernt Seizinger
1986	Ulrich Förstermann
1986	Edgar Schömig
1986	Martin Lohse
1988	Susanne Ott
1988	Markus Schwaninger
1990	Martin Feelisch
1990	Christopher Reithmann
1992	Claudia Kohl
1992	Stefan Offermanns
1994	Stefanie Dimmeler
1994	Heike A. Wieland
1996	Monika Stoll
1998	Andreas Friebe
1998	Ulrich Rümenapp
2000	Annette Nicke
2000	Stefan Engelhardt
2004	Rachel Jurd
2006	Henriette Meyer zu Schwabedissen
2008	Carolin Daniel
2009	Karin Eichele
2009	Annette Heinrich
2011	Andrea Ahles
2013	Andreas Bock
2019	Stephan Künzel
2019	Elias Rawish
2020	Lukas Menges
2021	Brit Silja Rohr
2021	Lukas Prüser
2021	Rachana Eshwaran
2022	Birte Niemann
2023	Konstantin Hennies
2023	Karin Ziegler

To determine whether the winners have succeeded in a scientific career we looked at their occupation and if they have become a professor. As the Fritz-Külz-Award is an award at the beginning of one’s scientific career, we took in account the latency from the award to possible professorship thus only analysing prize winners until 2008 (allowing at least 16 years latency). Until 2008, there have been 32 prize winners of which 26 (82.25%) became professors (this includes all types of professorship including full professor, associate professor, assistant professor and extraordinary professor). Three have not become a professor (two men, one woman), three could not be pursued long after the award, thus no information is available about their occupation (one man, two women). Of the 26 prize winners that have become professors so far, 19 were male (73.08%) and 7 were female (26.92%).

**Fig. 6:**
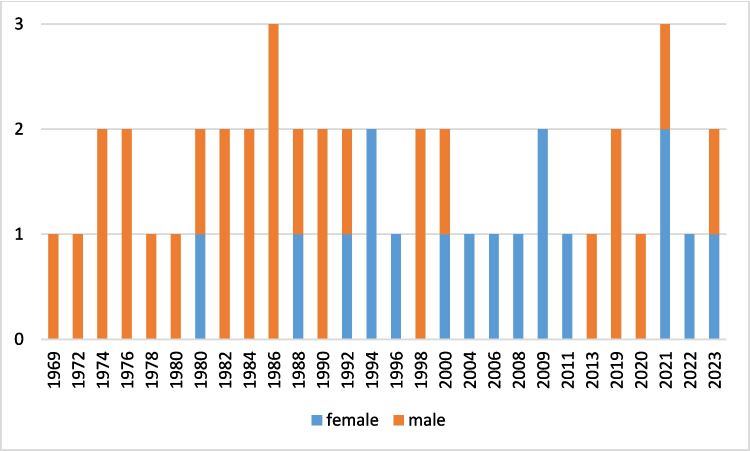
Fritz-Külz-Award 1969–2023. Number of prize-winners by gender

The data show that starting in the mid-1990s, more and more women were awarded the Fritz-Külz-Award which trend is in agreement with the trends observed for medical societies in general (Fig. 6).

**Fig. 7:**
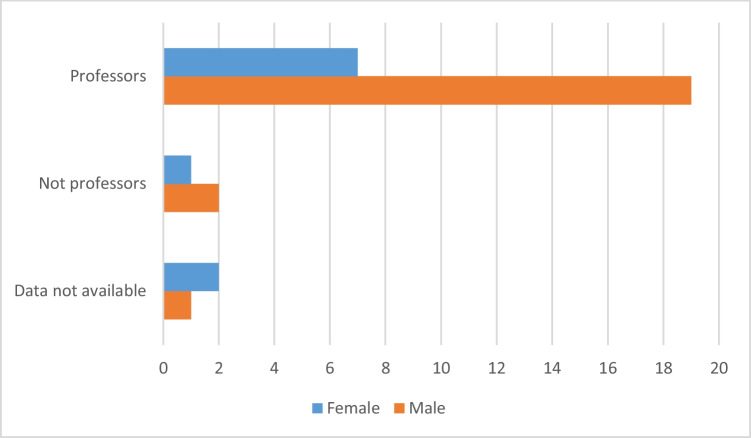
Distribution of occupation of the prize winners of the Fritz-Külz-Award by gender

The Fritz-Külz-Award targets junior pharmacologists having just completed their doctoral thesis. The award committee aims at selecting the most promising scientists with a potential of an academic career. The data reveal that, indeed, the award fulfils this mission since the majority of the awardees later obtained academic positions. The example of the Fritz-Külz-Award shows, in a nutshell, how important awards for junior scientists can be in terms of fostering the determination for an academic career (Fig. 7).

The composition of the committee making the decisions on the Fritz-Külz-Award has changed over the years. Previously, the Editors of Naunyn-Schmiedebergs Arch Pharmacol made the decisions, with the Editor-in-Chief having the largest influence. Some years ago, the review panel was modernized: Currently, the chairpersons and vice-chairpersons of the German Pharmacology Society (DGP, Deutsche Gesellschaft für Pharmakologie), the German Society for Clinical Pharmacology (DGKliPha, Deutsche Gesellschaft für Klinische Pharmakologie) and the Society for Toxicology (GT, Gesellschaft für Toxikologie), the Editor-in-Chief of Naunyn-Schmiedebergs Arch Pharmacol and the Executive Director of the DGPT jointly make the decisions. Currently, the committee comprises seven persons, ensuring that even in case of a close call, a majority can be achieved, avoiding a tie. All applications are first assessed and ranked individually by the committee members, i.e. the members receive the application portfolios and an assessment sheet. Members will have 3–4 weeks for assessing the applications. In case of conflict of interest, committee members must abstain from voting. In the next step, the ranking of applications is revealed anonymously to all committee members by the Executive Director, and the applications are discussed in a committee meeting. To reflect the diversity of the discipline, quite often two or even three prizes are awarded (Fig. 6). Currently, the Fritz-Külz-Award committee is composed of two women and five men. This composition is a reflection of the fact that in pharmacology and toxicology, women are still underrepresented in senior positions (Zehetbauer et al. 2022) and, hence, in positions qualifying for senior executive board positions at the society and editorial board level. Overall, the recently implemented modern awarding procedure has resulted in highly consensual decisions.

## Study limitations

Our study has only focused on German prizes in medicine, and not all medical societies have contributed data. Future studies aim at including more countries and other disciplines.

## Conclusions

Based on the assumption formulated at the beginning that prizes can contribute to the visibility of researchers, our study results suggest that the visibility of female scientists in German medicine has increased overall in the last 20 years. This finding is in line with the international studies, which did not focus exclusively on the most prestigious awards (Atkinson et al 2019). Overall, however, a gender award gap is still recognisable in the awarding of prestigious honorary prizes and higher-endowed research prizes in medical societies in Germany.

Differences between the specialist societies, depending on the speciality, the respective proportion of female specialists and the proportion of female scientists among the members must be investigated in follow-up studies. Furthermore, the factors and conditional relationships according to and in which prizes are established and awarded must be analysed (Malik et al. 2024, Bünemann and Seifert 2024). Since data on the composition of the prize committees is only available for a small proportion of the prizes and usually only for the current award, no statistical statements can be made on possible influences on the discrimination of women in the prize-awarding process for our study corpus. In order to be able to investigate structural discrimination against female scientists, the scientific societies would have to create more transparency in the data on nominated candidates and applicants (Lagisz et al 2024). For the Fritz-Külz-Award, we have revealed the awarding mechanisms as paradigm how transparency can be achieved. What is the gender distribution among the nominees? How many women apply for prizes? What is the composition of the committees? (Graves and Brashear 2018). It would be easier to address the fundamental consideration of parenting and caring periods in age restrictions.

## Data Availability

All source data for this work (or generated in this study) are available upon reasonable request.
